# Erythromycin Releasing PVA/sucrose and PVA/honey Hydrogels as Wound Dressings with Antibacterial Activity and Enhanced Bio-adhesion

**DOI:** 10.22037/ijpr.2019.1101002

**Published:** 2020

**Authors:** Shahrzad Fathollahipour, Mojtaba Koosha, Javad Tavakoli, Susan Maziarfar, Jalil Fallah Mehrabadi

**Affiliations:** a *Department of Chemical and Biomolecular Engineering, The University of Akron, 200 East Buchtel Common, Akron, OH 44325, USA. *; b *Faculty of New Technologies Engineering, Shahid Beheshti University, Tehran, Iran. *; c *Mechanical Engineering Biomechanics and Implants Research Group, The Medical Device Research Institute (MDRI), School of Computer Science Engineering and Mathematics, Sir Eric Neal Building, Flinders University, Adelaide, Australia. *; d *Faculty of New Science and Technologies, Department of Life Science Engineering, University of Tehran, Tehran, Iran. *; e *The Lister Laboratory of Microbiology, Tehran, Iran.*

**Keywords:** Erythromycin, Honey, Hydrogel, Polyvinyl alcohol, Sucrose, Wound dressing

## Abstract

The present study deals with preparation and characterization of thermally crosslinked PVA-based hydrogels containing honey and sucrose for the purpose of erythromycin delivery. The hydrogels have been characterized and compared by scanning electron microscopy, Fourier transform infrared spectroscopy, and bio-adhesion tests. Swelling measurements showed that addition of sucrose and honey decreased the equilibrium swelling of the hydrogels. Results of release studies showed that the amount of erythromycin, released at the early hours was higher for PVA/sucrose and PVA/honey hydrogels compared to PVA hydrogel while the drug released at later times was highly reduced for PVA/honey hydrogel. Both Peppas-Sahlin and Korsmeyer-Peppas models fitted well to the release data. Fitting Peppas-Sahlin model to the release data showed that at the initial times, release of drug from the hydrogel network was mainly governed by Fickian mechanism; however, at later times the drug is dominantly released by relaxational mechanism due to swelling of the network,. Addition of honey improved the bio-adhesion of PVA/honey hydrogel as compared to PVA/sucrose and pure PVA hydrogel. Results of antibacterial tests showed growth inhibitory action of erythromycin-loaded PVA hydrogels against *Pseudomonas aeruginosa* and *Staphylococcus aureus* bacteria. This study indicates that these hybrid hydrogels are capable of being used as functional wound dressings aiming to control the rate of antibiotic delivery to the wound site and prevent the wounds from infection.

## Introduction

Skin is the primary protective barrier of the human body against the external environment. Thus, any loss of integrity in this barrier might result in significant disability and could even lead to death. There are some cases when the structural integrity of skin is compromised by factors such as surgical procedures, infectious diseases, or certain pathological conditions. These types of circumstances are referred to as wounds and are in need of urgent and appropriate therapeutic strategies. Wound healing, as a dynamic biological process, is divided into five major interrelated stages of hemostasis, inflammation, migration of epithelial cells, proliferation, and maturation (1, 2).

The large diversity in wound types has given rise to a wide range of wound dressings and novel therapeutic strategies, the goal of which is to enhance the overall wound healing process. In this regard, there exist some medicated dressings, usually made of biopolymers, incorporated with therapeutics to overcome the disadvantages and side effects of topical drug administration. These therapeutic materials are usually within the groups of vitamins, growth factors, debriding and antibacterial agents. In an ideal condition, a desirable wound dressing should have the ability to maintain the damaged environment moisturized, while protecting the wound from secondary infections, reducing the risk of wound recurrences, absorbing the wound exudates and malodor, as well as having good mechanical properties, proper adhesion to the wound bed and easy removal (3-5). 

Polymeric wound dressings are mostly produced in form of films, hydrogels, hydrocolloids, foams, and nanofibers (1, 6, 7). With regard to the hydrogels, the features of moist wound healing come together with unique fluid absorption. Furthermore, the transparency of most hydrogels allows for the observation of healing stages with no need for removing the hydrogel (8). The hydrogel network has the ability to expand through secrete absorption and empty network spaces are responsible for absorbing foreign bodies such as bacteria, exudates, and odor molecules. Moreover, hydrogels are of great ability to encapsulate active materials within their network and release them to the injured area in a sustained and prolonged manner, which makes them candidates as functional cutaneous drug delivery systems (4, 9-13). 

Erythromycin, as a functional antibiotic agent, is a widely used macrolide antibiotic for the treatment of wound bacterial infections caused by gram-positive bacteria (14, 15). However, it has a short half-life (about 1.4–2 h) with a fast distribution rate all over the body (16). In this regard, controlled delivery of erythromycin through a hydrogel wound dressing can be effective for both preventing the wound site from infections, as well as protecting erythromycin from fast degradation and distribution. Drug-eluting hydrogels have been the subject to many studies so far. In a previous study, our group successfully synthesized gelatin nanospheres loaded with erythromycin, as a controlled-release antibiotic delivery system (17). We also prepared a nanocomposite drug releasing wound dressing nanofibers containing gelatin nanoparticles loaded with erythromycin (18). Both of gelatin nanoparticles and the nanocomposite wound dressing showed effective inhibitory action against *Pseudomonas aeruginosa* and *Staphylococcus aureus* bacteria. In another study, polyvinyl alcohol (PVA) film was utilized as a transdermal carrier for delivery of erythromycin in acne treatment (19). Among all the additives tested, poly (ethylene glycol) and glycerin were suitable for the preparation of erythromycin-loaded PVA films.

Hydrogels based on natural saccharides are also found to be appealing for wound healing applications due to their ability of being easily processed in hydrogel synthesis (21 and 22). Many studies have focused on the use of natural saccharides and disaccharides, such as honey and sucrose (natural sugar), to promote the wound healing process. Moore and coworkers investigated the topical use of honey in superficial wounds. The results showed the advantages of honey in treatment of such wounds (23). In a report by Wang et al., hydrogel sheets of chitosan, honey, and gelatin have been successfully used as burn wound dressings, and proved effective against *Staphylococcus aureus* and *Escherichia coli* and also in enhancing the healing process (24). 

In our previous work, we prepared erythromycin loaded PVA/honey hydrogels crosslinked with different concentrations of borax with a high concentration of honey and sustained release of erythromycin (2). In this research, we evaluated the physicochemical properties of erythromycin-loaded thermally crosslinked PVA, PVA/sucrose, and PVA/honey hydrogels. We hypothesized that the addition of sucrose and honey can affect the release profile of erythromycin and bio-adhesion of the hydrogel films. To the best of our knowledge, physically cross-linked PVA hydrogels containing sucrose, honey, and erythromycin have not been studied so far in the literature. Morphology, swelling behavior, and bio-adhesion of the hydrogels were measured and compared. The release of erythromycin from hydrogels was determined in pseudo extracellular fluid (PECF) media as the simulating wound fluid. In addition, the antibacterial activity of the wound dressings against *Pseudomonas aeruginosa* and *Staphylococcus aureus bacteria *was also tested by the inhibition zone method.

## Experimental

*Materials*

PVA (M_w_=145,000; >99% hydrolyzed) was purchased from Sigma-Aldrich (USA). Sucrose and ethanol were obtained from Merck chemicals (Germany). Erythromycin was purchased from AFA Chemie pharmaceutical company (Tehran, Iran). Natural honey was obtained from herbal medical store. All other chemicals were of analytical grade and used without any further purification.

*Synthesis of the hydrogels*

Aqueous solution of PVA with a concentration of 10% (w/v) was prepared by constant stirring at 100±2 °C at 1400 rpm for 2 h. Honey and sucrose were separately mixed with distilled water to achieve final concentrations of 15% (w/v). Solutions containing 1.5% (w/w) of each saccharide (honey and sucrose) in PVA were subsequently prepared via stirring at 1000 rpm for one hour at 80 °C to get a homogeneous hybrid mixture. Equal weights of PVA, PVA/sucrose, and PVA/honey mixtures were cast into Petri dishes and dried at room temperature for 48 to 72 h and respectively named PVA, PVA-S, and PVA-H through the text. To achieve physically cross-linked hydrogels, the samples were thermally treated in an oven at 80 ± 2 °C for a period of 12 h. With regard to the loading process, appropriate amounts of a 10% solution of erythromycin, in ethanol 70%, were added to the PVA/ sucrose and PVA/honey solutions to reach the solutions containing 1 wt% of erythromycin, followed by casting in Petri dishes. These samples were named PVA-E, PVA-S-E, and PVA-H-E, respectively. To avoid drug degradation as a result of applying temperature, the drug-containing hydrogel films were heated at 50±2 °C for a period of 24 h to achieve physically cross-linked hydrogels. This temperature was selected based on the literature (25). All samples were stored in desiccators for further use. 

*Swelling measurements*

Hydrogel networks were assessed in terms of their swelling properties in double distilled water and pseudo extracellular fluid (PECF). PECF solution which simulates wound fluids was prepared by dissolving 0.68 g of NaCl, 0.22 g of KCl, 2.5 g of NaHCO_3_ , and 0.35 g of NaH_2_PO_4_ in 100 mL of distilled water. The pH of pseudo extracellular fluid was fixed at 8.0 ± 0.5 (26, 27).

Swelling of the hydrogels was studied by gravimetric method as a function of the two parameters of saccharide type and temperature. Hydrogel films of the known dry weights were immersed in the double distilled water at 25 °C and taken out; the excessive liquid at the surface of the swollen films was removed using a filter paper and then weighed immediately in determined intervals until reaching a constant weight. Swelling characteristics of hybrid hydrogels were studied for different films of PVA-H, PVA-S, and pure PVA film. The swelling ratio (%S) of each sample was calculated using Equ. 1:

$\left(\%\right)S=\frac{W_{t}-W_{0}}{W_{0}}$                     Equ. (1)

Where, *W*_t_ is sample weight after swelling and *W*_0_ is its initial weight in the dry state. The same method was used to study the effect of temperature on the swelling ratio of the hydrogel films at 37°C and 40 °C using PECF as the swelling medium.

The swelling behavior of the samples at room temperature can be fitted using the following models. Model I (Equ 2) was fitted to the entire data while model II was fitted to the first 60% of water uptake data.

Model I S = S_e _(1- exp(-Kt)                      Equ. (2)

In model I, S is the swelling ratio of the sample at time t, S_e_ is the equilibrium swelling ratio and K is the kinetic constant (min^-1^). (28).

*Scanning electron microscopy (SEM)*

The surface of the hybrid hydrogels was observed via scanning electronic microscope. The samples were first double coated with a thin layer of gold to induce conductivity via a sputter coater unit. Subsequently, the surface characteristics of the samples were analyzed by a CamScan MV2300 Scanning electron microscope (UK).

*Fourier transform infrared spectroscopy (ATR-FTIR)*

The ATR-FTIR spectra of the dry films were recorded by a Thermo Nicolet 5700 (USA) FTIR system. The spectra were used to find out any possible formed bonds as a result of chemical interactions between the components. 

*Bio-adhesion studies*

**Figure 1:**
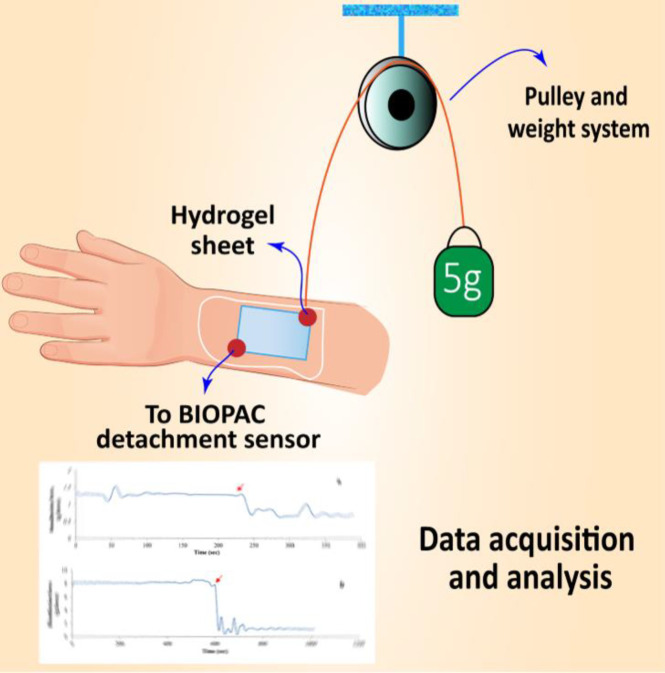
Schematic representation of the system used for the bioadhesion test

Bio-adhesion testing of the hydrogel films was carried out using the Biopak MP System and analyzed using Acknowledges software package (USA) as mentioned in the previous works (2, 29). Briefly, the objective of the system is to measure the amount of the time needed to detach the swollen hydrogel thin film from the human skin. Initially, the samples of known dimensions were immersed in double distilled water for 20 min in order to reach to the equilibrium state of swelling. After removing the excessive surface water, the hydrogel films were placed on the surface of human skin. The system was calibrated and a 5-g weight was attached to one side of the sample while the other side was hooked up the BIOPAC sensor using a string of known length. The system then measures the adhesive strength of the sample, the time needed for the sheet to detach from skin surface. This time index can be used as a credible measure to compare the adhesive strength of the samples. Results are reported as gram-force vs. time diagrams. The system used for bioadhesion test is presented in figure 1.

*Release studies of the antibiotic agent*

Release studies were performed by UV absorption method. To determine the wavelength at which the erythromycin shows its maximum UV absorption (λ_max_), a scan was performed over the entire range of UV-Vis. The specific wavelength where the drug showed the maximum UV absorption over the range was determined and used throughout the study. The indicative wavelength of erythromycin was recorded to be at 255 nm. In order to evaluate the release characteristics of erythromycin, several concentrations of erythromycin in ethanol 70% were prepared and the linear calibration curve was obtained and used for further studies. *In-vitro* release of the erythromycin was studied by placing a piece of dried and drug loaded film with a dimension of 2×2 cm^2^ in a standard jacketed Franz static diffusion cell filled with 100 ml PECF at 37 °C. A standard dialysis membrane (Mw =12KDa, Sigma Aldrich, USA) was placed between the sample and release media. The amount of erythromycin released in the medium was detected spectrophotometrically by measuring the absorbance of the sample solutions picked out of the diffusion cell in the fixed time intervals. The sink conditions were met by replacing the amount of the solution removed from the cell by the fresh PECF of the same volume in all time intervals. The kinetics of the drug release was evaluated by fitting model II (Korsmeyer-Peppas) and model III (Peppas-Sahlin) (Equ. 4 and Equ. 5) to the experimental data:

 Model II $\frac{M_{t}}{M_{\infty }}=kt^{n}$                      Equ. (3) (30)

 is the cumulative amount of the drug released at time t and is the cumulative amount of the drug released at infinite time, is the fractional drug release from the sample at time t, k is the constant characteristic of the hydrogel and exponent n is the mode of transport. This equation can be fitted to the first 60% of the drug release curve regardless of geometry of the carrier. Model III was chosen according to Peppas and Sahlin as following: 

Model III $\frac{M_{t}}{M_{\infty }}=K_{1}t^{m}+K_{2}t^{2m}$                     Equ. (4) (31)

In this model, the exponent m is the purely Fickian diffusion exponent of the device with any geometrical shape. The value of m should be used regarding the aspect ratio of the device, 2a/l, where 2a is the diameter and l is the thickness of the device. For 2a/l < 0.1, m = 0.45 and for 2a/l > 100, m = 0.5. In this work, we used 2×2 cm^2^ films with a thickness of ~0.1 mm, though the aspect ratios of the films were greater than 100 and we used m = 0.5 for fitting. In this model, the first term in the right hand side is the Fickian contribution and the second term is Case II relaxational contribution. According to Peppas and Sahlin, the ratio of relaxational (R) to Fickian (F) contributions can be calculated as:

$\frac{R}{F}=\frac{k_{2}}{k_{1}}t^{m}$                     Equ. 5

## Antibacterial Activity

The strains of standard *Pseudomonas aeruginosa* (ATCC 27853) and *Staphylococcus aureus* (ATCC 25923) were utilized for the evaluation of antibacterial activity of the wound dressings using the disc diffusion method. For each of the bacterial strains, a bacterial suspension was prepared from fresh colonies, the turbidity was adjusted to 0.5 McFarland standard (1×10^8^ CFU/mL) and incubated for 24 h at 37 °C. The suspensions were cultured on Muller-Hinton agar plates. The samples were punched into discs with 1 cm diameter, sterilized with UV radiation for 20 mins, placed in culture plates and incubated at 37 °C for 24 h. To control and compare the antibacterial activities of the samples, Ciprofloxacin (5 µg) standard discs were placed in each plate and the inhibition zones for the samples and the control discs were measured and analyzed. The data were obtained from 3 replicates. The diameter of the inhibition zone for each sample was measured using ImageJ software.

## Results

*Surface morphology of the drug-loaded hydrogel matrices*

**Figure 2:**
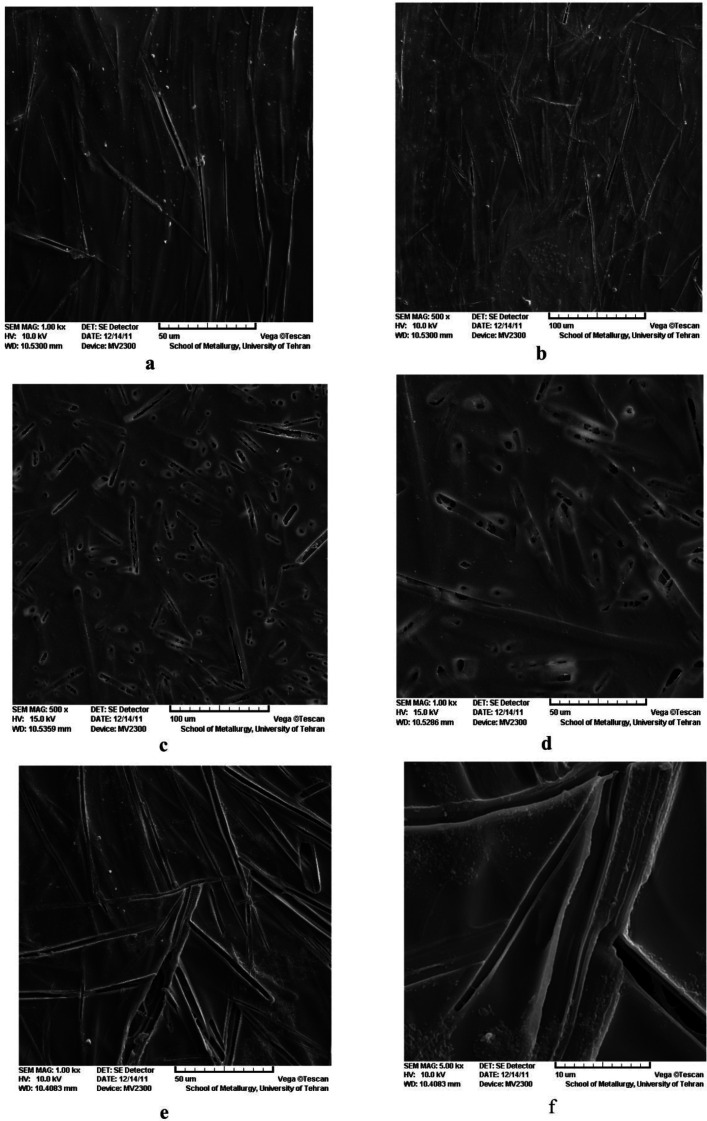
SEM images of the drug containing hydrogel films PVA-E: (a) 1000X, (b) 500X, PVA-S-E: (c) 5000X, (d) 1000X and PVA-H-E: (e)1000X, (f) 500X

The SEM micrographs of drug-loaded PVA hydrogels are shown in Figure 2A-F. The SEM micrographs showed two distinct morphological patterns. The first one, observed in PVA-H-E and PVA-E films (Figure 2A-B and Figure 2C-D), showed a needle-like surface structure for erythromycin aggregation in the film. Erythromycin was concentrated more in needle-shaped areas than in other parts. Erythromycin has a hydrophobic structure which is not compatible with hydrophilic PVA matrix. Though, they aggregate into needle like structures distributed in the PVA matrix. Aggregation could be attributed to the high surface energy of these structures. The needle-like structures appear relatively unidirectional. The second type of morphological pattern was observed in case of PVA-S-E (Figure 2 E-F), where the porous needles-like structures were not arranged in the same direction, unlike the first pattern. The resemblance of the structures observed in the two groups is clearly a result of their similar chemical structures. 

## Results of ATR-FTIR

**Figure 3:**
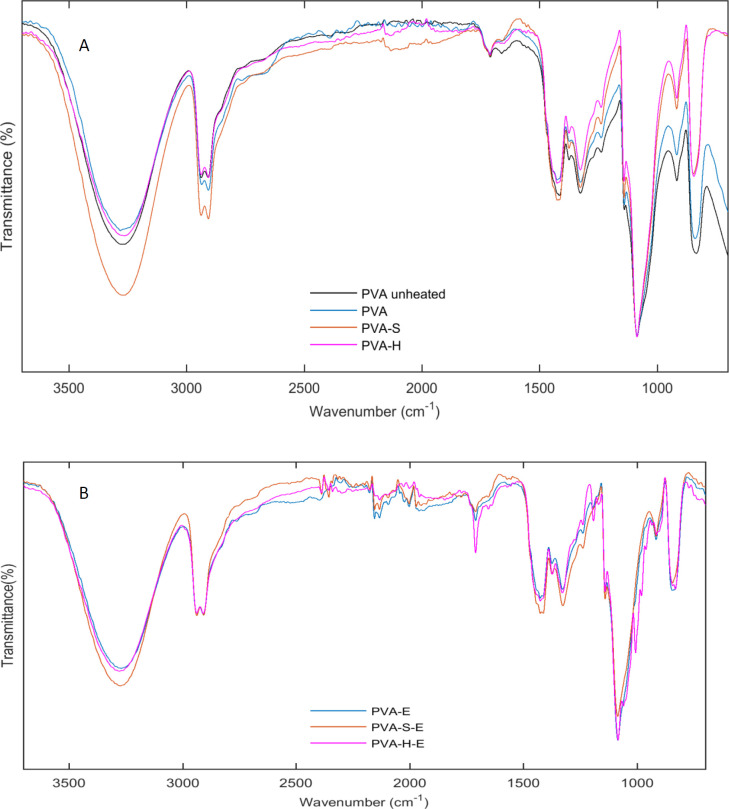
ATR-FTIR spectra for (a) PVA unheated, PVA, PVA-S, PVA-H and (b), PVA-E, PVA-S-E, PVA-H-E hydrogel films

ATR-FTIR spectra of hydrogel PVA membranes as well as drug loaded hydrogels are shown in Figure 3 A,B. In order to show the effect of heat treatment on the chemical structure of PVA, the ATR-FTIR spectra for the unheated and heated PVA samples were recorded. The broad band observed at 3265 cm^-1 ^in the spectrum of unheated PVA (Figure 3-A) is due to the stretching vibrations of hydroxyl groups. However, this peak is shifted to ~3278 cm^-1^ for thermally treated PVA hydrogel and its intensity is reduced after heat treatment. This result indicates that number of free hydroxyl groups is reduced after heat treatment. Heat treatment causes the PVA chains to form more crystalline structures. As PVA chains are hydrogen bonded in their crystalline structures, the more crystallinity leads to the reduction of free hydroxyl groups. The intensity of the hydroxyl groups is increased for PVA-S and PVA-H hydrogels compared to PVA sample. Addition of sucrose and honey adds an extra amount of solvent (water) to the PVA solution and thus, reduces the concentration of PVA chains leading to lower intensity for hydroxyl groups. The chemical structure of sucrose and honey is comprised of glucose and fructose rings linked together with acetal bonds. Honey is mainly composed of monosaccharides such as glucose, fructose, and galactose and a small amount of disaccharides such as maltose and sucrose. The main groups present in the structure of the mentioned saccharides are OH, CH2, and C-O groups which are similar to the chemical groups present in the structure of PVA. Though, the vibration bands of the chemical groups of sucrose and honey, overlap with that of PVA and we did not observe extra bands for PVA-S and PVA-H samples (32, 33). This result indicates the proper compatibility between sucrose, honey, and PVA though hydrogen bond formation between hydroxyl groups. However, PVA-H hydrogel had a lower intensity comparing to PVA-S. This result indicates more hydrogen bonding formation between hydroxyl groups of PVA chains and mono and/or disaccharides of honey.

The bands at 2910 and 2941 are due to vibrations of CH_2_ groups. Small peaks at 1710 cm^-1^ were observed for all samples which is due to carbonyl groups vibrations. Another small peak at 1660 cm^-1^ is probably related to presence of water molecules in the structure of hydrogels because the films were not heated in vacuum oven. The other bands appeared at 1425, 1327, 1142, 1086, 916, and 845 cm^-1^ are assigned to CH_2_ bending vibrations, CH, and OH bending vibrations, C-C and C=O stretching, C-O stretching, CH_2_ rocking and C-C stretching vibrations, respectively (34, 35). 

In case of erythromycin loaded hydrogels, similar peaks were observed for the samples. However, the peak at 1711 cm^-1^ appeared sharper and stronger for the drug containing samples. Erythromycin has two carbonyl groups in its chemical structure which causes this peak to appear more strongly in the drug containing hydrogels. Also, the bands appeared at 960, 979, 1005, 1244 cm^-1^ are present in the spectrum of the drug containing samples which were absent in the hydrogels without the drug. These results confirm the presence of erythromycin in the hydrogel samples.

*Swelling behavior*

*Matrix swelling as a function of saccharide type*

**Figure 4:**
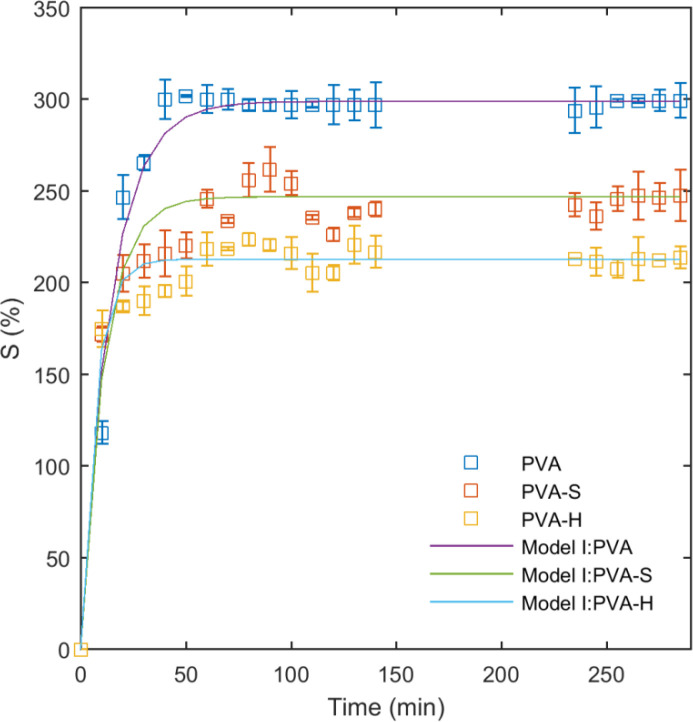
Swelling ratios of the hydrogel films as function of time in water at 25 ºC and the fitted model I to the data

The effect of saccharide type on the swelling characteristics of PVA/saccharide hydrogels was studied in double distilled water as shown on Figure 4. The initial swelling ratio after 10 minutes was recorded as 118%, 172%, and 174% for PVA, PVA-S, and PVA-H hydrogels, respectively, as measured by Equ. 1. It appears that PVA-S and PVA-H samples have a higher rate of water uptake at initial stages of swelling. This could be explained by the presence of water-soluble molecules of sucrose and honey in the network, playing a role in enhancing the penetration of water molecules into the hydrogel. However, by increasing the time, PVA network swells more than PVA-S and PVA-H samples and reaches a higher final equilibrium (Figure 4). The reduction of equilibrium swelling for PVA-S and PVA-H is related to the lower density of PVA chains in PVA-S and PVA-H samples due to the incorporation of sucrose and honey. Similar results have been reported previously for agar-incorporated PVA hydrogels elsewhere (36). It is worth mentioning that the effect of honey on swelling of the hydrogels was more than sucrose because of its higher molecular weight. From the swelling curves, the swelling behavior of the hydrogel networks before reaching the equilibrium can be divided into two distinct areas. In the first zone, from the initial measurement up to 20 minutes, the network swells with a higher rate. The second area extends from 20 to 40 minutes, where the average rate was decreased. 

The results of the fitting model I (Equ. 2) to the swelling data are presented in Table 1 and the curves are plotted on data in Figure 4. As can be seen from the curves and the obtained R^2^ values, this model fits well to the entire swelling data. In model I, K is the kinetic parameter which shows how quickly the polymer network reaches to the equilibrium swelling. For PVA hydrogel, K is small but it has increased to higher values for PVA-S and PVA-H samples. It means that PVA-S and PVA-H samples have reached to equilibrium swelling more quickly than PVA. It is also evident from the swelling curves. The equilibrium swelling ratio was lower for PVA-S and PVA-H compared to PVA and this helps the network to reach the equilibrium more quickly.

*Matrix swelling as a function of temperature*

**Figure 5:**
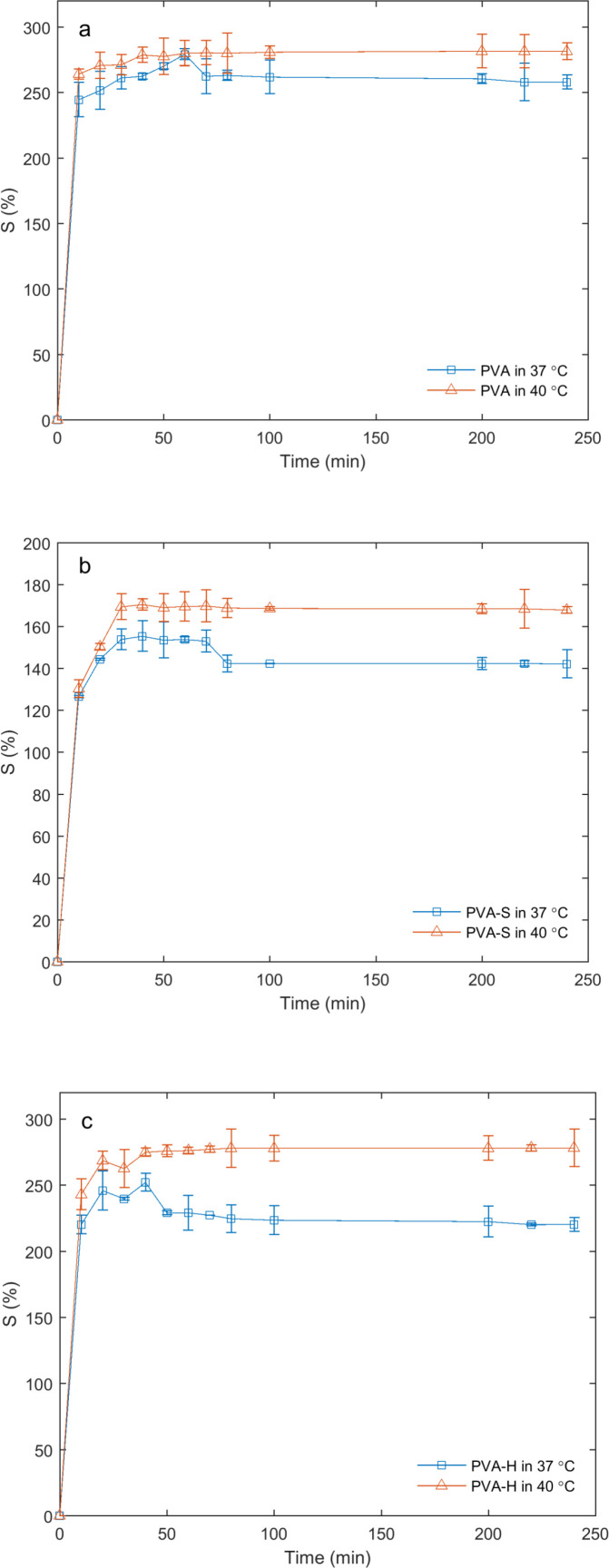
Swelling ratios of PVA (a), PVA-S (b) and PVA-H (c) hydrogels in PECF at the temperatures of 37 ºC and 40 ºC

Swelling studies as a function of temperature were carried out in simulated wound fluid (PECF) at 37 °C and 40 °C. As expected, the swelling ratio was increased with temperature rise (Figure 5 A-C) during the entire timescale of the experiment. It was observed that PVA-S and PVA-H samples were more sensitive to the temperature rise as compared to the PVA sample. 

*Drug release studies in simulated wound fluid*

**Figure 6:**
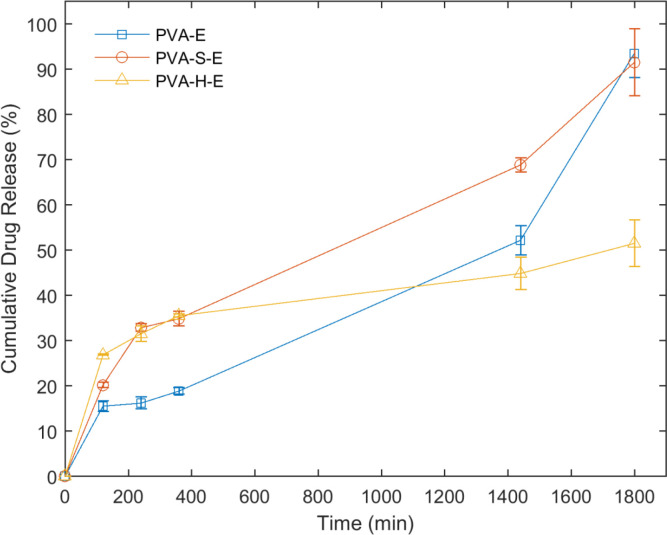
Cumulative release of erythromycin from hydrogels films in the PECF medium at 37 °C

The results of *in-vitro* release evaluation of erythromycin from the drug-loaded hydrogel networks are presented in Figure 6. The release of erythromycin from PVA-H-E hydrogel starts with the highest rate among other hydrogel samples. The next initial highest rates belong to the PVA-S-E and finally, PVA-E hydrogels. The amount of drug released in the first two hours of the experiment is considered as the burst release which has been less than 30% of the total amount of loaded drug in all hydrogel samples. The amount of drug released before 400 min is higher for PVA-S-E and PVA-H-E hydrogels but in later times, PVA-H-E sample has the lowest amount of drug released and an increase in the overall drug release was observed for PVA-E and PVA-S-E samples compared to PVA-H-E. This result indicates that presence of honey in PVA network can retard the release of erythromycin from the PVA matrix. Honey is composed to different saccharides with a dense viscous structure. When it is added to PVA, many hydrogen bonding interactions may occur between hydroxyl groups of PVA and saccharides. These physical interactions can prevent the drug diffusion out of the swollen matrix.

**Figure 7:**
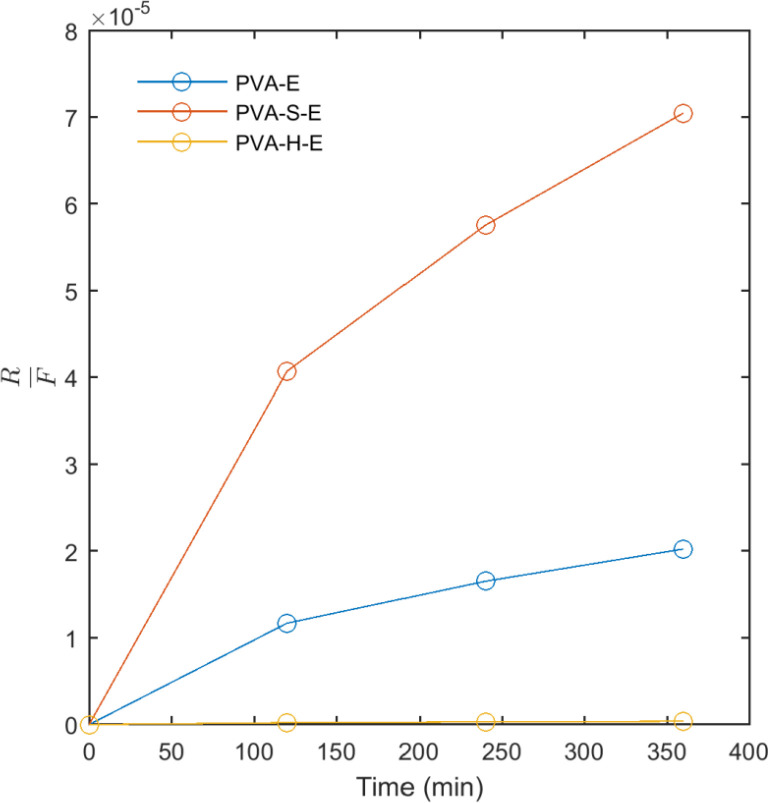
The ratio of relaxational to Fickian contribution (R/F) versus time obtained from fitting model III to the release data

The results of fitting model II (Korsmeyer-Peppas) and model III (Pepas-Sahlin) to the release data are presented in Table 2. These models were fit to the first 60% of the released mass of drug. The values obtained for R^2^ indicates that both models fit well to the release data. In model II, the release profile of an active agent from a matrix can be classified into Fickian (Case I) and non-Fickian (Case II) according to the n. In the Fickian (Case I) mechanism, n = 0.5 and the release of the drug is governed by diffusion. When n = 1, the mechanism is non-Fickian (Case II) and the release kinetics is zero order governed by the swelling or relaxation of polymer chains. When 0.5 < n < 1, the mechanism is non-Fickian or anomalous transport and the drug released by diffusion and swelling. According to Table 2, the obtained results for n in model II shows that for PVA-E hydrogel, the mechanism of drug release is non-Fickian governed by diffusion and swelling. For PVA-S-E, the mechanism is close to Fickian diffusion. However, the value of n obtained for PVA-H-E was lower than 0.5 and the mechanism of drug release cannot be explained by the obove classification. The parameters of model III were also reported in Table 2. From the data obtained for the parameters of this model, it can be seen that the values of K_1_ and K_2_ are nearly similar for PVA-E and PVA-S-E samples while these values are different for PVA-H-E hydrogel. As mentioned, K_1_ is the Fickian contribution and K_2_ is the relaxational contribution. So, we calculated the ratio of relaxational to Fickian contribution (R/F) as presented in Figure 7. This ratio was highest for PVA-S-E and then PVA-E and PVA-H-E. This result indicates that the mechanism of drug release is mainly relaxational for PVA-E and PVA-S-E hydrogels while Fickian diffusion is the predominant mechanism for PVA-H-E. The hydrogen bonding interactions between honey molecules and PVA chains act as physical crosslinks which prevent the drug diffusion outside the polymer network and result in a lower release rate. It is also notable that as shown in Figure 7, the ratio of relaxational to Fickian mechanism (R/F) is low at the initial times of release experiment for all samples while it is increased to higher values at later times. At initial times, the polymer network has not swelled completely and the mechanism of drug diffusion is mainly Fickian. At later times, the network has swelled to its equilibrium state and the drug diffusion out of the network is predominantly governed by relaxational motions of polymer chains in the network.

*Bio-adhesive strength*

**Figure 8:**
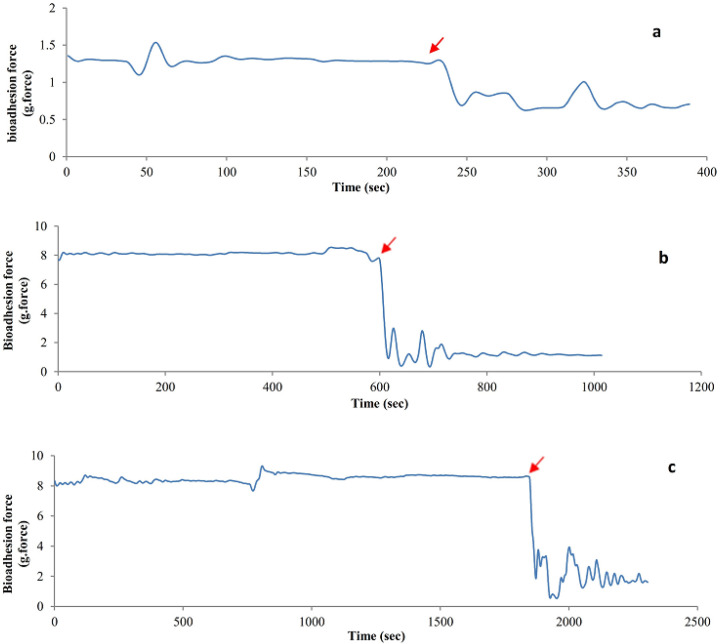
Bio-adhesive strength of drug containing swollen hydrogels of pure PVA (a), PVA-S (b) and PVA-H (c) obtained from the BIOPAC system

The results of the bio-adhesive strength of the hydrogel films are presented as detachment force-time diagrams (Figure 8 A-C). As it can be observed from the diagrams, the maximum detachment force and time needed for separation were different for the PVA, PVA-S, and PVA-H hydrogels. The sharp force drops in all diagrams is attributed to the time that the hydrogel has been detached from the skin surface. The more it takes for the hydrogel to detach from the skin surface, the better bio-adhesive properties it is believed to have. The detachment time is denoted in all diagrams of Figure 8. As obvious from the diagrams, PVA-H-E hydrogel showed the best adhesive strengths as it takes about 1800 seconds before its complete detachment from the skin surface. The detachment time was equal to 572 seconds and 231 seconds for PVA/sucrose and pure PVA hydrogel, respectively. The results show that the additions of the saccharides were helpful in enhancing the bioadhesive strength of PVA hydrogels.

*3.6. Antibacterial activity*

The antibacterial activity of the hydrogels against *Pseudomonas aeruginosa* and *Staphylococcus aureus *were evaluated and the size of inhibition zones was measured as reported in Table 3. Ciprofloxacin standard discs were placed as control on the plate to compare the inhibitory action of the samples. The PVA-S and PVA-H samples did not show any inhibitory effect on the bacteria. However, both of *Pseudomonas aeruginosa* and *Staphylococcus aureus bacteria *were sensitive to the Ciprofloxacin discs as well as PVA-S-E and PVA-H-E hydrogels. The hydrogels containing erythromycin showed an inhibition zone compared to the standard antibiotic disc. The difference between the sizes of the inhibition zones was not significant for the samples compared between the two bacteria (*p*-value> 0.1). Comparing this parameter between the samples shows that for *Staphylococcus aureus *bacterium the size of the inhibition zone for the ciprofloxacin disk was not different with PVA-E but different with PVA-S-E and PVA-H-E samples (*p*-value<0.001). In case of* Pseudomonas aeruginosa *the difference between the size of inhibition was not significant for the sample PVA-E and PVA-S-E compared to the control while the sample PVA-H-E had a significantly higher inhibition zone (*p*-value<0.002). This result reveals that the presence of honey and sucrose facilitates the diffusion of antibiotic through the hydrogel. During water absorption by the hydrogel, honey and sucrose leave out the hydrogel network by dissolution in the aqueous media. Thus, the hydrogels containing sucrose and honey would have more voids in their network comparing to PVA hydrogel which results in better diffusion of erythromycin out of the network and larger inhibition zone.

## Discussion

PVA hydrogels can be prepared by physical or chemical methods. The physical methods include freeze-thaw, irradiation, and thermal treatment (4, 36-39). In chemical methods, a crosslinking agent (e.g. glutaraldehyde, glyoxal, diisocyanate, etc) is used to react with PVA chains (40-43). Crosslinking PVA by physical methods hardly alters the biocompatibility of the hydrogels while in chemical methods, the unreacted crosslinking agents or their degradation products may cause cytotoxicity (44). The freeze-thaw method on the other hand, is time-consuming and needs several cycles to crosslink the PVA. However, PVA hydrogels can be prepared by thermal treatment. In this study, we prepared antibiotic-loaded PVA hydrogels for wound dressing application by thermal treatment. The samples without antibiotic were treated at 80 °C and those containing antibiotic were treated at 50 °C to prevent the degradation of erythromycin. Results of cell culture tests showed that the PVA hydrogels prepared in this study were biocompatible and did not show any cytotoxicity for the fibroblast cells (data not shown). The cells were attached to the surface of the hydrogels and proliferated to a normal morphology.

Swelling measurements in water showed that the addition of sucrose and honey in the hydrogels alters their swelling characteristics. This could be related to the high solubility of honey and sucrose in the liquid medium. The rates of solvent uptake in PVA-H and PVA-S hydrogels were higher in the initial stage (slope of the curves in Figure 4), followed by a gradual decline possibly attributed to the formation of higher intramolecular hydrogen bonds between sucrose and honey molecules which causes the excessive saccharides to dissolve in water and exit the hydrogel network. The swelling behavior of the samples in PECF was altered by increasing the temperature. The slight increase in kinetic energy may rearrange the hydrogen bonds between PVA and saccharides leading to new solvent–network bond augmentation which directly affects the swelling ratio variation. Furthermore, it is notable that solubility of some saccharides in the solvent increases with temperature which forces the saccharides to leave the hydrogel network. Following the saccharide immigration, the open spaces in the hydrogel network will be filled with the solvent. Immediately after PECF penetrates into the hydrogel, the membrane swells and part of the drug content entrapped in the network can be washed by PECF medium, taking the dissolved drug out of the network. The described phenomenon mostly occurs in the surface areas of the swollen network where the transition from a glassy to a rubbery state takes place for PVA chains due to the presence of water molecules. The release of drug through the entire hydrogel network was closely related to their swelling characteristics.

Application of the drug-loaded hydrogels prepared in this study, in a real situation as a wound dressing material can be described as follows. As in the initial stages of wound healing, the wound area has the most liquid content, the hydrogel wound dressings would be able to absorb the wound exudates and replace it with sufficient amount of antibiotic to prevent the possible infections. After a while, in the second phase, PVA would be the single controlling agent of the drug release and the risk of infection decreases as a result. In this stage, there would be a proportional reduction in the amount of drug released from the dressing.

In all of the samples, the starting rate of erythromycin release has been higher than the later stages. This can be attributed to the equilibrium swelling ratio of the networks. In the first stage, it is better to deliver a constant amount of drug to the wound area in order to avoid any infection (here in the first 240 minutes). The diffusion of PECF molecules is easier through the polymeric chains and thus the swelling rate is faster in the initial stages. In contrast, in the later stage, while reaching the equilibrium, the degree of swelling decreases dramatically. As the slope of the line approaches zero, it can be concluded that the drug delivery system is optimal. 

**Table 1:** Results of fitting model I to the swelling data in water at 25 °C

	*Model I (Equ. 2)*		
Sample code	*S*_e_	*K (min* ^-1^ *)*	*R* ^2^
**PVA**	2.99	0.071	0.980
**PVA-S**	2.47	0.09	0.943
**PVA-H**	2.13	0.146	0.963

**Table 2:** Results of fitting model II (Korsmeyer-Peppas) and model III (Peppas-Sahlin) to the release data

	Model II			Model III		
Sample code	*n*	*k*	*R* ^2^	*K*_1_	*K*_2_	*R* ^2^
**PVA-E**	0.615	0.005	0.979	0.01082	1.15×10^-^^8^	0.924
**PVA-S-E**	0.471	0.022	0.983	0.01933	7.18×10^-8^	0.983
**PVA-H-E**	0.224	0.092	0.993	0.02023	4.11×10^-10^	0.960

**Table 3:** Results of antibacterial activity against Staphylococcus aureus and Pseudomonas aeruginosa of the hydrogels compared to the standard ciprofloxacin disc

Sample code	Inhibition Zone (mm)	
	*Staphylococcus aureus*	*Pseudomonas aeruginosa*
**Ciprofloxacin disc**	25.9±1.9	28.1±0.5
**PVA**	0	0
**PVA-S**	0	0
**PVA-H**	0	0
**PVA-E**	16.9±0.9	15.4±1.9
**PVA-S-E**	27.0±2.2	23.1±4.3
**PVA-H-E**	28.9±4.5	26.2±3.8

## Conclusions

We have successfully developed and reported the PVA/sucrose and PVA/honey hydrogel networks loaded with erythromycin as an antibiotic agent. The ATR-FTIR spectra proved the existence of the drug in the network and suggest no new harmful bonds between the samples. The addition of honey and sucrose improved bio-adhesive properties comparing to the pure PVA hydrogel. The release of antibiotic was highly reduced by addition of honey to the hydrogel. Fitting kinetic models to the release data showed that the kinetics of drug release is mainly Fickian in the initial times and changes to relaxational mechanism in the later times of release due to swelling of the network. The erythromycin loaded PVA hydrogels showed a notable inhibitory action against *Pseudomonas aeruginosa* and *Staphylococcus aureus* bacteria. The overall results indicate the potential application of these drug-eluting PVA-hydrogel transparent sheets as highly effective and practical wound dressings.
